# Outcomes of DISE-Directed Surgery for Obstructive Sleep Apnoea in Children

**DOI:** 10.3390/children12091185

**Published:** 2025-09-05

**Authors:** Rachel Blokland, Yael Friedland, Aryan Kalra, Adelaide Withers, Shyan Vijayasekaran

**Affiliations:** 1 Department of Otolaryngology, Perth Children’s Hospital, Nedlands, WA 6009, Australia; rachelblokland@gmail.com (R.B.); aryan.kalra@health.wa.gov.au (A.K.); 2 Department of Respiratory and Sleep Medicine, Perth Children’s Hospital, Nedlands, WA 6009, Australia; 3 Medical School, University of Western Australia, Crawley, WA 6009, Australia

**Keywords:** DISE, paediatric OSA, adenotonsillectomy, polysomnography, tongue-base reduction

## Abstract

**Highlights:**

**What are the main findings?**

- DISE-directed surgery improved PSG outcomes in younger, non-obese children and those with severe pre-operative OSA.
- Tongue-base reduction had the highest rate of improvement among DISE-guided interventions, while children with Trisomy 21 showed limited benefit.

**What is the implication of the main finding?**

- DISE allows for targeted, individualised surgical planning in complex paediatric OSA cases, with better outcomes in specific subgroups.
- Children with multiple comorbidities, Trisomy 21, or obesity may require alternative or adjunctive approaches beyond DISE-directed surgery.

**Abstract:**

**Background:** Obstructive sleep apnoea (OSA) affects 1–4% of children and may cause significant health issues if left untreated. While adenotonsillectomy is the primary intervention, up to 33% of children experience residual OSA. Drug-induced sleep endoscopy (DISE) enables identification of the site of anatomical obstruction, thus facilitating targeted surgical interventions. The approach of the institution at which this research was conducted is to only perform DISE in selected patients including those who fail adenotonsillectomy or when adenotonsillectomy is not expected to be successful. **Methods**: This retrospective case series reviewed 19 children who underwent DISE and DISE-directed surgeries for OSA at Perth Children’s Hospital between 2018 and 2021. Polysomnography (PSG) parameters pre- and post-surgery were compared. **Results**: Overall, there were no significant improvements in PSG parameters in the group post-surgery. However, improvements were found with sub-group analyses in younger children, those with severe pre-operative OSA and non-obese children. No significant improvement was observed in children with Trisomy 21. Revision adenoidectomy was the most frequently performed surgery and tongue-base reduction achieved the highest rate of improvement (80%) based on PSG parameters. **Conclusions**: DISE-directed surgery offers a promising approach for managing residual paediatric OSA. Outcomes may be favourable in younger patients, those with severe OSA, and non-obese patients. Further research with larger cohorts is warranted to refine surgical strategies.

## 1. Introduction

Obstructive sleep apnoea (OSA) is common in children, affecting between 1 and 4% of children in the United States. If left untreated, OSA may lead to growth retardation, poor school performance, behavioural problems, and mood disturbance [1].

Adenotonsillectomy has been shown to improve mood, learning, and behavioural problems in most children affected by OSA. However, depending on other factors, 15 to 33% of children will have residual OSA following adenotonsillectomy. High-risk groups for surgical failure include syndromic children, those with craniofacial anomalies, obesity, and those with severe OSA [2].

The diagnosis of OSA is made with polysomnography (PSG) [1,3]. However, PSG cannot identify the anatomical site of airway obstruction [4]. Drug-induced sleep endoscopy (DISE) has been shown to be able to identify the site of anatomical obstruction and therefore can guide surgical intervention [5]. The patient is sedated to mimic natural sleep after which a flexible nasendoscope is used to visualise dynamic airway collapse during respiration. DISE was first described by Croft and Pringle in 1991 [6].

A meta-analysis published in 2019 assessed the outcomes of airway surgery guided by DISE and Cine Magnetic Resonance Imaging (CineMRI) or computed tomography (CT) scan in children with residual OSA after adenotonsillectomy. Overall, there was a reduction in the apnoea hypopnea index (AHI) of events by 6.5/h and an improvement of the lowest oxygen desaturation (LSAT) by 3%. The authors concluded that DISE or imaging-directed surgery for OSA results in a significant improvement in AHI and LSAT [7].

The primary outcome of this study was to assess changes in PSG parameters—specifically the Obstructive Apnoea–Hypopnea Index (OAHI)—following DISE-directed surgical intervention in children with OSA. Secondary outcomes included identifying clinical predictors for improvement, such as age, body mass index (BMI), severity of OSA, and comorbidities like Trisomy 21, as well as assessing the frequency and effectiveness of the different DISE-directed procedures performed.

This study focuses on a high-risk population of children, including those with comorbidities and prior surgical intervention, in the hopes of better understanding the role of DISE in complex paediatric OSA.

## 2. Methods

### 2.1. Study Design

A retrospective cohort study with chart review was undertaken. This study was conducted in accordance with the Declaration of Helsinki (as revised in 2013). This study was approved by the ethics board of the Children’s and Adolescent Health Service (CAHS) Clinical Governance Unit (Governance Evidence Knowledge Outcomes (GEKO) Quality Activity #46967), and individual consent for this retrospective analysis was waived. The data collection process adhered to ethical guidelines, ensuring confidentiality and anonymity of all individuals involved. The STROBE reporting guideline, accessed online through Enhancing the QUAlity and Transparency Of health Research (EQUATOR), was used in manuscript preparation.

### 2.2. Subjects

Children who underwent DISE at Perth Children’s Hospital between 2018 and 2021 were identified for inclusion. DISE is only performed at this institution in selected patients—typically those with persistent OSA following adenotonsillectomy or those in whom adenotonsillectomy is unlikely to be successful. Patients were excluded if they did not undergo surgical intervention after DISE or lacked both pre-operative and post-operative PSG for comparison. Of the 24 children who underwent DISE during the study period, 19 met the inclusion criteria and formed the final study cohort. All 19 patients in our study received surgical interventions during the same anaesthetic as the DISE.

### 2.3. Polysomnography

The PSGs were performed in the National Association of Testing Authorities, Australia (NATA) accredited Sleep Disorders Unit at Perth Children’s Hospital according to unit protocols, American Academy of Sleep Medicine and Australasian Sleep Association/Australasian Sleep Technologists Association recommendations [8,9,10], and published clinical practice guidelines [11,12,13] with Compumedics™ Profusion PSG 3 equipment (Compumedics™ Limited, Victoria, Australia). Type 1 PSGs were attended overnight by sleep scientists and measured electroencephalography (EEG), electrooculography (EOG), chin and diaphragmatic electromyography (EMG), airflow with nasal pressure and oro-nasal thermistor, respiratory effort with inductance plethysmography, electrocardiography (ECG), pulse oximetry, transcutaneous carbon dioxide monitoring (TCO_2_), body position, snore volume, and time-linked audio-video. Although all raw data was available for scoring scientists and reporting sleep physicians to view, only the total sleep time, mean and nadir oxygen saturations, number of apnoeas/hypopnoeas, and OAHI were collected from the PSG report for analysis. Sleep scientists used standard rules [8,9,14] to stage sleep and score respiratory events, and reports were generated by paediatric sleep physicians. An acceptable PSG was defined by technical success (i.e., whether signals were available and interpretable) and a total sleep time of more than or equal to 6 h. No minimum amount of rapid eye movement (REM) sleep time was specified for an acceptable study.

The changes in OAHI and oxygen saturation nadir pre- and post-surgery were compared. Severity of paediatric OSA was defined based on OAHI values as follows: mild (1–5 events/h), moderate (5–10 events/h), and severe (>10 events/h) as per American Academy of Sleep Medicine Guidelines (AASM) [11].

### 2.4. Drug-Induced Sleep Nasendoscopy

All DISE procedures were performed at one primary institution by multiple surgeons from the same Otorhinolaryngology (ENT) department. At Perth Children’s Hospital, there is no fixed anaesthetic protocol for this procedure; however, the general guidelines are to avoid any premedication and topical anaesthesia to the larynx, and nasal vasoconstriction should be delayed until after the nasal airway has been adequately assessed. For children who tolerate awake intravenous (IV) cannulation, dexmedetomidine and propofol were used: dexmedetomidine initial loading dose of 0.5–1 µg/kg, followed by infusion of 0.5–1 µg/kg/h; propofol is administered via Target-Controlled Infusion (TCI) using the Paedfusor model with a target of 2 µg/kg/min. Children who do not tolerate awake IV cannulation are given an induction with Sevoflurane to facilitate IV cannulation. Once IV access is established, cease Sevoflurane and initiate dexmedetomidine and propofol as outlined above. Patients were positioned in a supine position. A 2.8 mm flexible endoscope was then passed to assess the turbinates, adenoid, palate, tonsils, oropharynx, tongue base, epiglottis, and supraglottis. This was performed with and without jaw thrust manoeuvre to assess the difference in airway patency between the two states. Results were recorded using a combination of descriptive methods and the VOTE grading system, which assesses obstruction at the level of the vellum, oropharynx, tongue base, and epiglottis (VOTE) [15].

### 2.5. Data Analysis

We first analysed the full cohort to assess differences between pre- and post-operative PSG results. Improvement was defined as a reduction in median OAHI of ≥50% following surgery in keeping with the Sher criteria.

Subgroup analyses were then performed. Patients were divided into two groups: those who showed improvement in post-operative PSG findings and those who did not. Differences between these groups were evaluated to identify predictors of response to DISE-directed surgery. In addition, we examined whether any demographic or clinical factors were statistically associated with post-operative improvement in PSG results.

Descriptive statistics were used to summarise the data. Normality of quantitative variables was assessed using the Shapiro–Wilk test. Parametric data (e.g., age) were presented as mean ± standard deviation (SD) and analysed using Student's *t*-test. Non-parametric data (e.g., OAHI, SpO_2_ nadir) were reported as median with interquartile range (IQR) and analysed using the Wilcoxon signed-rank test or Mann–Whitney U test, as appropriate. Fisher’s exact test was used for categorical comparisons. A *p*-value of <0.05 was considered statistically significant. All statistical analyses were performed using R Commander (version 4.2.3).

## 3. Results

### 3.1. Demographics

The cohort included 57.9% males (N = 11), with ages ranging from 23 days to 15.6 years (mean age 7.2 ± 4.5 years). Most patients (84.2%, N = 16) had at least one comorbidity, with Trisomy 21 as the most common (N = 6). Details of comorbidities are outlined in Table 1. Three patients were classified as underweight for age, and six patients were considered obese (BMI > 95th centile). In total, 12 of the 19 had prior adenotonsillectomy, 2 had prior adenoidectomy, 1 had prior tonsillectomy, and 4 were surgically naïve. Craniofacial abnormalities were the primary indication for DISE in the surgically naïve group, affecting three patients: one with Pierre Robin sequence (PRS) and cleft palate, one with micrognathia, and one with Patau syndrome.

### 3.2. Surgical Interventions

**Figure 1:**
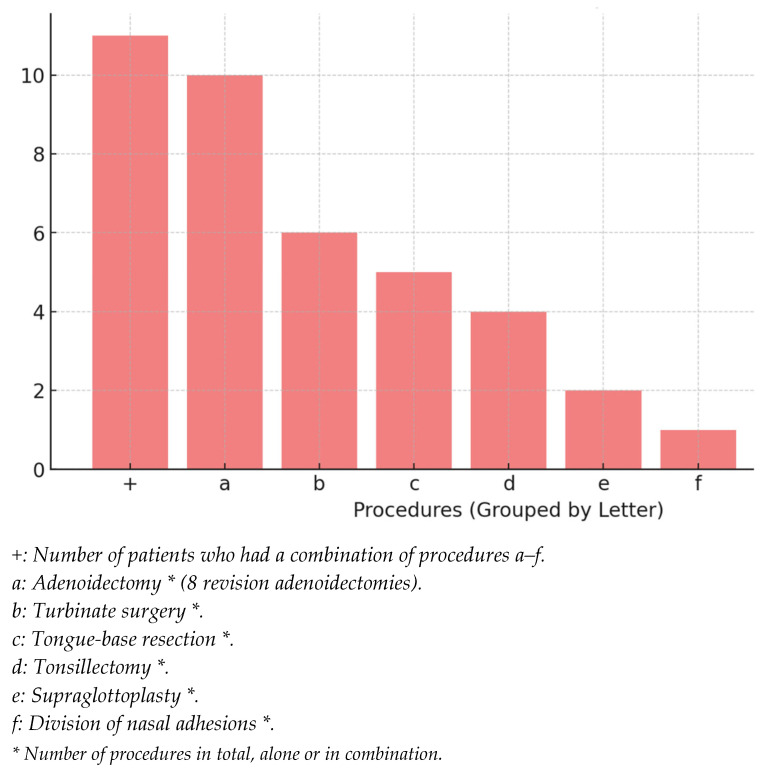
Frequency of DISE-directed procedures performed.

Surgical procedures were based on the DISE findings and performed during the same anaesthetic session. Both the DISE assessments and the subsequent surgeries were conducted by various surgeons from the same ENT department. Interventions included adenoidectomy (N = 10), turbinate reduction (N = 6), tongue-base procedures (N = 5), tonsillectomy (N = 4), supraglottoplasty (N = 2), division of nasal adhesions (N = 1), and laryngeal type 1 cleft repair (N = 1) (Figure 1).

1. Adenoidectomy (N = 10, 52.6%)

Of the ten patients who underwent adenoidectomy, nine underwent adenoidectomy in conjunction with other procedures: five with turbinate procedures, three with tonsil surgery, and one with tongue-base reduction surgery. Two were primary adenoidectomies, and eight were revision procedures. Of the primary adenoidectomies, one was performed alone in a six-month-old baby who was ex-premature and had micrognathia, and the other was performed as part of primary adenotonsillectomy in a 2-year-old child.

2. Turbinate reduction (N = 6, 31.6%)

Only one patient had turbinoplasty alone, while the other five were performed in conjunction with revision adenoidectomy.

3. Tongue-base reduction (N = 5, 26.3%)

Most of the tongue-base reductions were performed in isolation, while one was performed with a revision adenoidectomy.

4. Tonsil surgery (N = 4, 21.1%)

Three of the four tonsil procedures were performed with revision adenoidectomy, and one was in isolation. The tonsillectomy performed without adenoidectomy was in a child with Trisomy 13 and a cleft palate.

5. Supraglottoplasty (N = 2, 10.5%)

One supraglottoplasty was performed in a patient with PRS who also had placement of a nasopharyngeal airway, and the other was performed in conjunction with a repair of type 1 laryngeal cleft in a patient with Ehlers–Danlos Syndrome.

6. Division of nasal synechiae (N = 1, 5.3%)

This was performed as the sole procedure in an 11-year-old patient with Opitz GBBB syndrome.

### 3.3. Outcomes

Improvement after surgery was defined as a reduction in OAHI of more than or equal to 50% on PSG.

When analysing the whole patient study population (N = 19), there were no statistically significant changes to pre- and post-intervention OAHI (6.3 vs. 2.9, *p* = 0.14, 95% CI (−0.7, 9.8)) or LSAT (85.0 vs. 86.0, *p* = 0.82, 95% CI (−6.5, 6.0)) following DISE-directed surgical intervention.

With sub-group analysis, using the Wilcoxon signed rank test, younger age, higher severity of OSA, and the absence of a diagnosis of Trisomy 21 were found to be predictors of improvement. Obesity was associated with failure to improve post-operatively.

Of the 19 patients, 10 met the criteria for improvement. Children who showed improvement were significantly younger than those who did not (mean age 3.6 vs. 10.7 years, *p* = 0.0001, 95% CI: −10.5 to −3.6). A higher pre-operative OAHI was also associated with improvement (median 10.1/h vs. 3.9/h, *p* = 0.04) (see Table 2).

On subgroup analysis, patients without Trisomy 21 showed a significant reduction in median OAHI following DISE-directed surgery (10.1/h to 2.2/h, *p* = 0.018, 95% CI: 1.65 to 18.15). Similarly, patients with severe OSA (pre-operative OAHI > 10/h) showed a significant improvement (14.2/h to 4.0/h, *p* = 0.03, 95% CI: 1.0 to 20.9) (see Table 3 and Table 4).

Children with a BMI below the 95th centile also benefited from surgical intervention as opposed to those above the 95th centile. In the non-obese group, median pre-operative OAHI was 8.2/h vs. a post-operative score of 2.6/h, Table 5 (*p* < 0.02). In patients with a BMI > 95th, there was no improvement in OAHI.

It should be noted that subgroup sizes were small, which may limit the strength of comparative analysis and generalisability of these findings.

Adenoidectomy (alone or as part of a multilevel approach) was the most commonly performed surgery in the patient cohort (N = 10, 52.6%). Of these, eight were revision procedures, and two were primary adenoid surgeries. Six out of the ten adenoidectomies (including both primary procedures) showed improvement in post-operative PSG parameters.

Tongue-base reduction was the procedure with the highest rate of improvement, with four out of five children showing a reduction in OAHI in their post-procedural PSGs (80% vs. 60% for adenoidectomy).

Turbinate surgery was the second most frequent DISE-directed intervention with six children undergoing this procedure. Tonsillectomy was performed in four of our patients and supraglottoplasty in two. Each of these procedures resulted in a 50% rate of improvement in OAHI.

Division of intranasal adhesions and a type 1 laryngeal cleft repair were each performed in one patient, but neither made a difference to the PSG results post-operatively.

## 4. Discussion

This study demonstrates that DISE can guide surgical decision making in a complex paediatric population with OSA, enabling targeted, multilevel interventions during the same anaesthetic session. Our findings suggest that DISE-directed surgery can result in objective improvements in OSA severity, particularly in younger, non-obese children with severe preoperative OSA and without Trisomy 21. At our institution, DISE is reserved for selected patients—those with persistent OSA following adenotonsillectomy or in whom initial surgery is unlikely to succeed. As a result, our cohort was characterised by a high level of complexity: 84.2% had one or more comorbidities or syndromic diagnoses. The majority had undergone at least one prior upper airway surgery before DISE-guided intervention: 12 of the 19 had prior adenotonsillectomy, 2 had prior adenoidectomy, 1 had prior tonsillectomy, and 4 were surgically naïve.

The high comorbidity burden among our cohort contrasts with several previously published cohorts reporting lower rates of comorbidities: 0% in Esteller et al., 10% in Saniasiaya et al., 35% in Socarras et al., and 57.6% in Wootten et al. [7,16,17,18]. However, He et al. described a cohort more comparable to ours, with 98% of patients having at least one comorbidity, including laryngomalacia (36.3%), Trisomy 21 (36.3%), developmental delay (34.5%), reactive airway disease (18.2%), and Pierre Robin sequence (9.1%), all having undergone prior upper airway surgery [19]. Estellar et al. also noted that, while none of their patients had comorbidities, all had a history of prior surgery [16]. In a systematic review by Saniasiaya et al., only 10% of 996 patients had a comorbidity or syndrome [17]. Socarras et al. reported a 35% comorbidity rate among 120 DISE patients [7], while Wootten et al. found that 54% of their 26 patients had Down syndrome, with the rest being non-syndromic [18]. A slight male predominance was observed in our study (57.9%), which aligns with findings from He et al. (66%) and Esteller et al. (70%). Other studies showed more balanced distribution of sex, such as Wootten et al. (50% male) and Socarras et al. (53.5% male) [7,16,17,18,19].

Studies assessing DISE-directed surgery use different outcome measures, with pre- and post-operative PSG comparisons being the most common [7,16,17,18,19,20,21]. Rate of change in surgical plan after DISE is also sometimes quoted [17,20], as are subjective measures such as improvement in snoring using visual analogue scale [16] and symptom burden [18]. While several prior studies demonstrated significant improvements in PSG parameters—such as OAHI and lowest SpO_2_ nadir—our study did not find statistically significant improvements across the entire cohort. This may reflect our small sample size but also likely relates to the greater clinical complexity of our patients, as comorbidities such as developmental delay and craniofacial anomalies are known risk factors for surgical failure [22]. Our data support the efficacy of certain DISE-directed procedures. Tongue-base reduction achieved an 80% rate of improvement, aligning with a systematic review by Sim et al., which showed that DISE-guided tongue surgery reduced AHI by 50% and improved LSAT by 3% [21]. This aligns with previous research from our team, where midline posterior glossectomy led to OSA resolution in 10 out of 15 patients (67%), with a 100% cure rate in the 7 children of normal weight [23].

Adenoidectomy was the most frequently performed surgery in our cohort (52.6%), consistent with He et al. (48%), and showed a 60% rate of improvement in our sample. Tonsil surgery and tongue-base procedures occurred at similar rates between studies (27% vs. 21.1%, and 20% vs. 26.3%, respectively), although supraglottoplasty was less common in our group (10.5%) than in He et al.’s cohort (38%) [19].

Considering most of the procedures were performed as part of a multilevel approach in our series, it cannot be accurately determined which individual procedure or combination of procedures was responsible for the improvement in OAHI.

Other studies differed in surgical distribution. Wootten et al. reported tongue-base surgery as the most common procedure, while Esteller et al. found that tonsillectomy was most frequently performed. However, revision adenoidectomy was common across most studies [16,18]. Our data suggests that age, severity of OSA, and a diagnosis of Trisomy 21 influence surgical response. Specifically, younger age and the absence of a diagnosis of Trisomy 21 are positive predictors of post-operative improvement in OAHI—this is in line with the existing literature [5,24]. However, our finding that children with higher pre-operative OAHI scores were more likely to benefit from DISE-directed surgery contrasts with the findings of He et al., who found that children with lower OAHI scores benefitted more [19]. In their study, patients who benefited from surgery had a pre-operative OAHI of 11.5/h, while those who did not had an OAHI of 19/h. Previous studies support this finding, although these were in children undergoing primary adenotonsillectomy [24].

This difference is interesting as the He et al. population was very similar to ours, and the types of surgery performed were also similar. However, in the He et al. cohort, less than 10% of cases underwent a midline posterior glossectomy, whereas, in our cohort, it was about 25%. We found that, in this complex group of patients, especially with high rates of Trisomy 21, where macroglossia is a common cause of severe OSA, midline posterior glossectomy was often associated with improvement. This may account for the difference.

Obesity is a well-established risk factor for severe OSA and reduced surgical response with adenotonsillectomy alone. In our cohort, patients with a BMI below the 95th percentile were more likely to benefit from DISE-directed surgery. This aligns with findings from Van de Perck et al., who studied primarily surgically naïve patients. They found that 50% of obese children had persistent OSA after DISE-directed adenotonsillectomy, which they attributed to the frequent finding of circumferential airway collapse. They found that these patients responded well to continuous positive airway pressure (CPAP) [25].

This study has several limitations. Its retrospective design limited analysis to objective PSG parameters and did not account for subjective outcomes such as sleep quality, symptom/quality-of-life improvements, or caregiver-reported changes. Some details of the PSG, such as sleep architecture data and extended oximetry parameters, were not included in our study. The small overall cohort and limited subgroup sizes reduce statistical power and may have obscured meaningful trends. Additionally, DISE assessments were performed by multiple surgeons without a standardised grading system, introducing variability in interpretation and surgical decision making. Finally, sedation techniques varied, and, although most patients received dexmedetomidine and propofol, the lack of a strictly protocolised approach could have influenced the DISE findings.

Despite these limitations, this study offers valuable insights into the role of DISE-directed surgery in managing complex paediatric OSA. Future research with larger, prospective cohorts and standardised protocols is essential to validate these findings and optimise surgical strategies.

## 5. Conclusions

DISE may be a valuable diagnostic tool for identifying anatomical sites of airway obstruction and guiding surgical intervention in paediatric patients with OSA.

Our study demonstrates that DISE-directed surgery can lead to improvements in objective PSG parameters in (1) younger children, (2) those with severe OSA, (3) those with a BMI less than the 95th centile, and (4) those without Trisomy 21.

Adenoidectomy and tongue-base reduction showed the most promising outcomes in our cohort, with the latter having the highest rate of improvement in PSG parameters.

Future research with larger, prospective studies and standardised protocols for DISE assessments is needed to refine surgical strategies and evaluate the clinical and subjective outcomes of DISE-directed interventions.

In conclusion, DISE is used adjunct in the management of paediatric OSA, particularly in cases of residual or complex airway obstruction. By identifying site-specific obstructions, it guides tailored surgical interventions, which may lead to improved outcomes for appropriately selected patients. However, further research is required to optimise its application and assess its long-term benefits.

## Figures and Tables

**Table 1:** Patient comorbidities and DISE-directed procedures performed.

Syndrome/Comorbidity	Number	Other Airway Factors	Surgeries Performed
Trisomy 21	6	2 patients with TOF * repair, 1 with laryngomalacia	Turbinoplasty 2× TBR ^$^ Revision adenoidectomy and CIT ^@^ Revision adenoidectomy and tonsillotomy Revision adenoidectomy and turbinectomy
FASD ^#^	1		TBR ^$^
Achondroplasia	1		Revision adenoidectomy, tonsillotomy
Rubinstein–Taybi Syndrome	1		Lingual tonsillectomy
Patau Syndrome	1	Cleft palate	Tonsillotomy
Opitz G/BBB Syndrome	1	Tracheobronchomalacia cleft palate	Division of nasal synechiae
Prader–Willi Syndrome	1		Revision adenoidectomy and TBR ^$^
Ehlers–Danlos Syndrome	1	Laryngeal cleft	Supraglottoplasty and repair of type 1 cleft
Pierre Robin Sequence (PRS)	1	Cleft palate	Supraglottoplasty and NPA ^§^ insertion
Asthma	1		Revision adenoidectomy and CIT ^@^
Ex-Preterm	1	Micrognathia	Adenoidectomy
None	3		2× Revision adenoidectomy and CITs ^@^ Adenotonsillectomy

* TOF: Tracheoesophageal fistula; ^#^ FASD: Foetal alcohol syndrome; ^$^ TBR: Tongue-base resection; ^@^ CIT: Cautery of inferior turbinates; ^§^ and NPA: Nasopharyngeal airway.

**Table 2:** Comparison of patients with and without improvement in OAHI after DISE-directed surgery.

	Patients Who Showed Improvement After DISE-Directed Surgery * (n = 10)	Patients Who Had No Improvement After DISE-Directed Surgery * (n = 9)	*p*-Value (95% CI)
Age (years ± SD)	3.6 ± 2.1	10.7 ± 4.1	0.001 * (−10.5, −3.6)
Comorbidities n (%)			
Trisomy 21 n (%)	1 (10.0)	5 (55.5)	0.057 (0.06, 0.76)
Prior adenotonsillectomy n (%)	6 (60.0)	9 (100)	0.09 (−0.03, 0.74)
Median BMI (IQR ^#^)	18.0 (5.3)	16.9 (2.8)	0.36 (−1.5, 9.5)
**Pre-operative PSG scores**			
Median OAHI (IQR ^#^)	14.2 (18.0)	4.0 (8.3)	0.03 * (1.0, 20.9)
Median of mean SpO_2_ (IQR ^#^)	97.0 (2.3)	97.0 (1.0)	0.27 (−3.0, 0.5)
Median SpO_2_ nadir (IQR ^#^)	83.5 (14.5)	90.0 (11.0)	0.22 (−16.0, 3.0)
Median VOTE score (IQR ^#^)	5.0 (2.5)	5.0 (2.0)	0.80 (−2.0, 1.0)

^#^ interquartile range * based on OAHI on PSG.

**Table 3:** PSG scores pre- and post-DISE-directed surgery in patients without Down syndrome (n = 13). The background colour indicates the column headings. The * indicates statistical significance. Bold indicates statistical significance.

	Median (IQR) Score Before DISE-Directed Surgery	Median (IQR) Score After DISE-Directed Surgery	*p*-Value (95% CI)
OAHI (events/h)	10.1 (13.8)	2.2 (6.4)	0.018 * (1.65, 18.15)
Mean SpO_2_ (%)	97.0 (1.0)	97.5 (2.0)	0.69 (−3.5, 1.0)
SpO_2_ nadir (%)	85.0 (13.0)	86.0 (17.0)	0.80 (−10.0, 10.0)

**Table 4:** PSG scores pre- and post-DISE-directed surgery in patients with severe OSA (n = 8). The * indicates statistical significance. Bold indicates statistical significance.

	Median (IQR) Score Before DISE-Directed Surgery	Median (IQR) Score After DISE-Directed Surgery	*p*-Value (95% CI)
OAHI (events/h)	18.5 (10.0)	7.0 (10.0)	0.039 * (3.9, 30.0)
Mean SpO_2_ (%)	97.0 (1.3)	98.0 (1.9)	0.35 (−7.0, 1.5)
SpO_2_ nadir (%)	74.5 (18.3)	81.0 (11.0)	0.74 (−24.5, 13)

**Table 5:** Patients with BMI < 95. The * indicates statistical significance. Bold indicates statistical significance.

	Pre-Operative Median (IQR)	Post-Operative Median (IQR)	*p*-Value (95% CI)
OAHI (events/h)	8.2 (12.3)	2.6 (8.7)	0.02 * (−15.3, −0.9)
Mean SpO_2_ (%)	97.0 (1.3)	97.0 (2.0)	0.82 (−1.5, 3.3)
SpO_2_ nadir (%)	86.0 (21.3)	88.0 (13.3)	0.61 (−7.0, 10.0)
OA (events/h)	7.5 (34.3)	5.0 (13.3)	0.14 (−53.5, 6.0)
MA (events/h)	0.0 (1.0)	0.5 (2.0)	0.73 (−3.0, 3.0)
CA (events/h)	9.0 (8.5)	5.0 (19.8)	1.0 (−4.0, 7.0)
OH (events/h)	44.0 (73.5)	17.0 (38.5)	0.045 * (−61.0, −1.5)
MH (events/h)	0.0 (0.0)	0.0 (0.0)	-
CH (events/h)	0.0 (0.0)	0.0 (1.5)	-

OA: obstructive apnoeas; MA: mixed apnoeas; CA: central apnoeas; OH: obstructive hypopnoeas; MH: mixed hypopnoeas; and CH: central hypopnoeas.

## Data Availability

The original contributions presented in this study are included in the article. Further inquiries can be directed to the corresponding author.

## Abbreviations

AASM	American Academy of Sleep Medicine Guidelines
AHI	Apnoea–Hypopnoea Index
BMI	Body Mass Index
CAs	Central Apnoeas
CAHS	Children’s and Adolescent Health Service
CHs	Central Hypopnoeas
CineMRI	Cine Magnetic Resonance Imaging
CITs	Cautery of Inferior Turbinates
CPAP	Continuous Positive Airway Pressure
CT	Computed Tomography
DISE	Drug-Induced Sleep Endoscopy
ECG	Electrocardiography
EEG	Electroencephalography
EMG	Electromyography
ENT	Ear, Nose, and Throat/Otorhinolaryngology
EOG	Electrooculography
EQUATOR	Enhancing the QUAlity and Transparency Of Health Research
FASD	Foetal Alcohol Syndrome
GEKO	Governance Evidence Knowledge Outcome
IQR	Interquartile Range
IV	Intravenous
LSAT	Lowest Oxygen Saturation
MAs	Mixed Apnoeas
MHs	Mixed Hypopnoeas
NATA	National Association of Testing Authorities, Australia
NPA	Nasopharyngeal Airway
OAs	Obstructive Apnoeas
OAHI	Obstructive Apnoea–Hypopnoea Index
OHs	Obstructive Hypopnoeas
OSA	Obstructive Sleep Apnoea
PRS	Pierre Robin Sequence
PSG	Polysomnography
REM	Rapid Eye Movement
SD	Standard Deviation
SpO_2_	Peripheral Oxygen Saturation
TBR	Tongue-Base Resection
TCO_2_	Transcutaneous Carbon Dioxide Monitoring
TCI	Target-Controlled Infusion
TOF	Tracheoesophageal Fistula
VOTE	Vellum, Oropharynx, Tongue Base, and Epiglottis
